# EXPLORING EARLY BARRIERS TO KIDNEY TRANSPLANT: THE MORAL ECONOMIES OF DIALYSIS CLINICS

**DOI:** 10.1093/geroni/igae098.3199

**Published:** 2024-12-31

**Authors:** Jenny McDonnell, Ellen Idler, Adam Wilk

**Affiliations:** Emory University, Atlanta, Georgia, United States; Emory University, Atlanta, Georgia, United States; Emory University, Atlanta, Georgia, United States

## Abstract

Kidney transplant conveys steep survival and quality of life benefits, yet only one-third of patients with kidney failure are referred for transplant evaluation. We explore the dialysis clinic as a site of inequities in transplant, wherein dialysis care professionals’ (DCPs’) early resource allocation decisions shape patients’ downstream access to transplant. Using a modified grounded theory approach, we conducted 39 in-depth interviews during summer 2022 with DCPs (e.g., nephrologists, social workers, nurses) about their processes leading to transplant referral or non-referral. Phone interviews were recorded and transcribed verbatim. Textual data were coded using MAXQDA software. Combining within- and across-case strategies for analysis, we uncovered tension between DCP’s reported referral philosophies and their tacit strategies for selectively allocating resources. While most DCPs reported that they “refer all patients”, in practice, they selectively direct limited resources (e.g., time, education, advocacy, empowerment) toward “ideal” patients. DCPs described “ideal” patients as compliant, activated, and motivated, compared to “risky” patients who “don’t do what they’re supposed to do”, struggle with self-management, and may “get less use out of a transplanted kidney” due to advanced age. We extend previous research by framing the dialysis clinic as a moral economy in which resources flow through a web of specific norms, meanings, and practices related to DCPs’ perceptions of patients’ deservingness and worth. Findings indicate that within this moral economy, DCPs direct limited resources toward “ideal” patients and away from “risky” patients most in need of DCPs’ time and advocacy. Calls for future research and policy implications are discussed.

